# Place de la chirurgie dans la prise en charge des cancers du sein chez la femme au Centre Hospitalier Universitaire Yalgado Ouedraogo: à propos de 81 cas

**DOI:** 10.11604/pamj.2015.22.117.6929

**Published:** 2015-10-12

**Authors:** Nayi Zongo, Timonga Françoise Danielle Millogo-Traore, Sidpawalmdé Carine Bagre, Abdoul-Halim Bagué, Edgar Ouangre, Maurice Zida, Aboubacar Bambara, Tozoula Augustin Bambara, Si Simon Traoré

**Affiliations:** 1 Département de Chirurgie, Service de Chirurgie Viscérale et Digestive, Centre Hospitalier Universitaire Yalgado Ouédraogo, Ouagadougou, Burkina Faso; 2 Service de Gynécologie Obstétrique, Centre Hospitalier universitaire Yalgado Ouédraogo, Burkina Faso; 3 Centre Hospitalier Universitaire Aristide Le Dantec, Institut Joliot Curie, Dakar, Sénégal

**Keywords:** Cancer, sein, chirurgie, Ouagadougou, Cancer, breast, surgery, Ouagadougou

## Abstract

Etudier la place de la chirurgie dans la prise en charge des cancers du sein au centre hospitalier universitaire Yalgado Ouédraogo. Nous avons réalisé une étude prospective et descriptive sur dix (10) mois portant sur la place de la chirurgie dans le cancer du sein. Elle a eu pour cadre les services de gynécologie-obstétrique et de chirurgie viscérale et digestive du centre hospitalier universitaire Yalgado Ouédraogo. Ont été pris en compte les indications, les gestes et les résultats de la chirurgie. Nous avons colligé 81 cancers mammaires. Le délai moyen de consultation a été de 14,26 mois. Les tumeurs T3 à T4 représentaient 82,71% des cas. Trente-huit patientes (46,91%) ont été opérées. La chimiothérapie néo adjuvante a été réalisée dans 29,63% des cas. Trente-quatre patientes (41,97%) étaient opérables d'emblée. Il s'agissait de mastectomie selon Madden dans 94,74% des cas et de chirurgie de propreté dans 2 cas (5,26% des cas). Une chimiothérapie adjuvante a été réalisée chez 52,63% des patientes opérées. Des complications à type de lymphocèle ont été notées dans 23,68% des cas. Leur traitement a consisté en des ponctions évacuatrices. Les indications de la chirurgie sont limitées par le retard diagnostique corollaire de stades avancés des cancers du sein. L'absence de la radiothérapie rend délicate la pratique de la chirurgie conservatrice et la mastectomie occupe toujours une place importante. Un diagnostic précoce permettrait d'augmenter les indications chirurgicales.

## Introduction

Le cancer du sein est une tumeur maligne de la glande mammaire [1]. C'est la première cause de décès par cancer chez la femme dans le monde [2]. Le dépistage systématique et les énormes progrès thérapeutiques ont permis un diagnostic précoce voire infra clinique et un pronostic meilleur dans les pays les mieux équipés et organisés [3]. Aux Etats Unis, le taux de survie à 5 ans qui était de 75,1% entre 1975 et 1977 est passé à 90% entre 2001 et 2007 [4]. Outre la chirurgie, la radiothérapie, l'hormonothérapie, l'immunothérapie, la chimiothérapie, les thérapies ciblées font partie de l'arsenal thérapeutique des cancers du sein [5]. Depuis sa première description par Halsted en 1891, la chirurgie mammaire s'est modernisée devenant moins agressive (Patey en 1948, Madden en 1972, la technique du ganglion sentinelle en 1994) tout en conservant son potentiel curatif [6]. Les traitements adjuvants et néo adjuvants ont permis à la chirurgie de conforter son rôle de traitement curatif dans le cancer du sein [6]. Une chirurgie adéquate permet de guérir 80% des cancers du sein au stade I [7]. Bien que le traitement des cancers du sein soit pluridisciplinaire, certains moyens thérapeutiques sont difficiles d'accès voire inaccessibles pour les pays en développement. Ce problème touche surtout les pays d'Afrique noire où la chirurgie est le traitement le plus accessible. Le Burkina Faso, pays pauvre de l'Afrique de l'ouest n’échappe pas à cette dure réalité. En pratique courante, les cancers d'emblée opérables semblent peu fréquents, les gestes chirurgicaux sont diversifiés, les indications parfois discutables. Cette étude a pour but de redonner à la chirurgie mammaire sa place dans la prise en charge des cancers du sein au Burkina Faso.

## Méthodes

Nous avons réalisé une étude prospective et descriptive. Elle a couvert la période du 1^er^ Mars 2013 au 31 Décembre 2013, soit dix (10) mois. Notre étude s'est déroulée dans le service de chirurgie viscérale et digestive du centre universitaire Yalgado Ouédraogo à Ouagadougou. Les services de gynécologie-obstétrique et le laboratoire d'anatomie pathologique furent sollicités. Toutes les patientes atteintes de cancer du sein histologiquement confirmés et ayant donné leurs consentements éclairés ont été incluses.

Les données ont été recueillies au cours d'entretiens directs avec les patientes. Les registres d'admission, les dossiers des malades ainsi que les cahiers de compte rendu opératoire ont servi également de source de données. Les patientes ont été vues avant leur inclusion dans l’étude, avant et après chaque traitement (chirurgie, radiothérapie, chimiothérapie), et de façon systématique tous les 3 mois jusqu′à la fin de l’étude ou au décès de la patiente.

En ce qui concerne les données générales, nous avons noté un âge moyen de 43,84 ans. Le délai moyen de consultation était de 14,26 mois avec des extrêmes de 14 jours et 30 ans. Le carcinome canalaire infiltrant était le type histologique le plus représenté avec 95,08% des cas. Le grade II et III représentaient respectivement 31% des cas et 48,81% des cas. Soixante-dix-huit patientes soit 96,3% avaient un statut performance inférieur ou égal à 2 selon la classification de l'OMS. La localisation métastatique était par ordre de fréquence le foie (11 cas), le poumon (9 cas), et l'os (3 cas). Cinq (5) patientes présentaient des localisations secondaires associant le foie, le poumon ou les os.

Ont été étudiés la proportion de malades ayant eu une chirurgie d'emblée, les indications et les gestes chirurgicaux, l'agencement des différentes thérapies (chirurgie, radiothérapie et chimiothérapie), les complications de la chirurgie. L'anonymat et la confidentialité des informations recueillis ont été respectés. En outre l’étude a eu l'autorisation du directeur général de l'hôpital, ainsi que des différents chefs des services qui nous ont servi de cadre d’étude.

## Résultats

Nous avons colligé 81 cancers mammaires. Le délai moyen de consultation a été de 14,26 mois. Les tumeurs T3 à T4 représentaient 82,71% des cas. Trente-huit patientes (46,91%) ont été opérées. Trente-quatre patientes de notre série soit 41,97% étaient opérables d'emblée. Seulement 31 de ces patientes ont été opérées. Il s'est agi de tumeurs de taille T1 (02 cas),T2(10 cas), T3(12 cas), T4a (02 cas), T4b(08 cas),T4c(02 cas). Les tumeurs de tailles T3 à T4 ont été opérées d'emblées par manque de moyens financiers pour réaliser une chimiothérapie néoadjuvante bien que cela fut indiquée. Les ganglions étaient cliniquement classés N0 (06 cas), N1 (20 cas) et N2 (07 cas). Toutes les patientes opérées étaient non métastatiques.

Les gestes réalisés étaient la mastectomie associé à un curage axillaire homolatéral dans 36 cas, une mastectomie de propreté dans 02 cas chez des patientes présentant une tumeur ulcéro-nécrotique d'environ 15-20 centimètres de grand axe, nauséabonde, adhérant au plan profond. Deux patientes opérables d'emblée ont été perdues de vue. Le traitement locorégional (mastectomie) a été refusé par une patiente de 48 ans qui ne voulait pas perdre son sein. Parmi les patientes non opérables d'emblée, 23 patientes soit 28,39% des cas ont bénéficié d'une chimiothérapie néoadjuvante. Le protocole FAC (5’-Fluoro uracile + Adriamycine + Cyclophosphamide) a été utilisé. Seulement 7 patientes ayant bénéficié de chimiothérapie néoadjuvante ont été opérées secondairement. Elles étaient vivantes à la fin de notre étude et ne présentaient aucune récidive locale. Le curage axillaire a consisté en l’évidement ganglionnaire des deux premiers étages de Berg. Il a été réalisé dans 36 cas.

Les limites du curage ont été pour toutes les patientes en dehors le bord antérieur du muscle grand dorsal, en dedans le nerf thoracique long (Charles Bell), en haut la veine axillaire, en bas la trifurcation de l'artère thoraco-dorsale, en arrière le pédicule du grand dorsal cheminant sur le muscle sub scapulaire. Le nombre moyen de ganglions au curage axillaire était de 11,15 ganglions. Nous n'avons noté aucun incidents per opératoires. L'examen anatomopathologique n'a pas été réalisé pour 6 pièces opératoires soit 15,79% des pièces opératoires. Le nombre moyen de ganglion histologiquement envahis (N+) était de 5,22 avec des extrêmes de 2 et 18 ganglions. Cinquante-trois virgule douze pour cent (53,12%) des cas présentaient un envahissement d'au moins 3 ganglions. Aucun patient n'a pu réaliser l'immunohistochimie.

Outre la chimiothérapie néo-adjuvante réalisée dans 23 cas, nos patientes ont eu en adjuvant une radiothérapie dans 2 cas, une chimiothérapie adjuvante dans 20 cas et une hormonothérapie dans 7 cas. Les patientes non opérables (22,22%) ont eu une chimiothérapie palliative dans 2 cas. Les suites opératoires immédiates ont été simples chez 36 patientes (94,74% des cas opérés). Des complications sont survenues chez 11 patientes (28,95%) dont 2 cas d'infection de la plaie opératoire survenue dans les suites de mastectomie de propreté. Une antibiothérapie et un pansement quotidien au dakin ont permis d'obtenir la cicatrisation dans tous les deux cas. Neuf patientes soit 23,68% des patientes opérées ont présenté un lymphocèle. Les lymphocèles ont été traitées avec succès par des ponctions répétées de façon aseptique.

Des métastases pulmonaires ont été notées dans un cas, 2,33 mois après la chirurgie. Une radiographie pulmonaire avait été réalisée. Un scanner thoracique aurait été plus contributif. Une patiente a présenté une récidive. Il s'est agi d'une tumeur de taille T4c n'ayant pas bénéficié de chimiothérapie néo-adjuvante et adjuvante par manque de moyen financier bien que cela fut indiqué. Le suivi moyen était de 4,73 mois. Douze (12) patientes soit 14,81% des cas ont été perdues de vue dont trois (3) patientes opérées. Le taux de décès des patientes opérées était de 5,26%.

## Discussion

Les stades avancés T3 ou T4 ont représenté la majorité des lésions avec 82,71% des cas. Ce constat a été aussi fait par la plupart des études africaines [8]. Le diagnostic tardif est donc l'une des caractéristiques des cancers du sein en Afrique. Cela pourrait s'expliquer par le retard à la consultation, le manque de campagne de dépistage, une sensibilisation insuffisante, l'absence de système de sécurité sociale. La prédominance des stades T3 ou T4 rend difficile voire impossible la réalisation d'une chirurgie conservatrice. Elle impose une mastectomie après une chimiothérapie néoadjuvante [9]. La chirurgie devient dès lors, le deuxième niveau thérapeutique malgré sa grande accessibilité dans nos contrées.

Par contre, grâce au programme de dépistage par la mammographie, les tumeurs de petites tailles T1 ou T2 constituent la majorité des lésions dans les pays développés [10, 11]. Elles représentaient 68% des cas dans l’étude de CUTULI [10] en France contre 32% de taille T3. GREENBERG [11] aux Etats Unis, retrouvait 78,5% de taille T1 ou T2. Dans ces situations, la chirurgie a conservé toute sa place curative. Elle est complété par la chimiothérapie et /ou la radiothérapie en adjuvant. Parfois la chirurgie constitue même le seul traitement [7, 10]. La majorité de nos patientes avait une taille tumorale supérieure à 5 cm donc justiciable d'une chimiothérapie néo-adjuvante. Le taux élevé de chimiothérapie néo-adjuvante a également été observé par d'autres études africaines [8, 12]. Le taux de chimiothérapie néo-adjuvante est en nette augmentation au Centre Hospitalier Universitaire Yalgado Ouédraogo [13]. Son augmentation considérable pourrait s'expliquer par l'insuffisance voire l'absence d'action visant à lutter contre le retard diagnostique. Fort est de constater que la chimiothérapie prend de l'ampleur au détriment de la chirurgie dans notre pays. Les indications chirurgicales d'emblée ont représenté moins de 50% des cas à cause des stades avancés. Les résultats de la chimiothérapie néoadjuvante sont largement en deçà des attentes. Seules sept sur 23 patientes ont eu une réponse à la chimiothérapie permettant de réaliser la chirurgie. Ceci s'expliquerait par l'irrégularité des cures voir leur interruption à cause du faible pouvoir d'achat et /ou de l'absence de subvention de la chimiothérapie dans notre contexte de travail.

Les avantages que confère la chirurgie dans les cas de tumeur de petite taille (T1, T2) ont été rapportés par plusieurs auteurs [7, 9]. Nous pensons que l'idéal serait de mener des actions pour un diagnostic précoce des cancers du sein. La place prépondérante de la mastectomie a été également rapportée par plusieurs séries africaines [8, 12]. Cependant, en occident elle ne représente que 30,5% des cas [10]. L'insuffisance de moyens financiers pour réaliser une chimiothérapie néo-adjuvante, l'irrégularité des cures, le diagnostic à un stade très avancé et l'inaccessibilité de la radiothérapie expliqueraient la forte proportion de la mastectomie dans notre série (45,68%). La chirurgie radicale modifiée (Madden) reste de ce fait le traitement le plus adéquat pour un contrôle de la tumeur primitive au détriment de la chirurgie conservatrice.

La chirurgie conservatrice prend de l'ampleur dans les pays industrialisés [10]. Le dépistage systématique par mammographie dans ces pays expliquerait sans doute cette avancée. En effet, elle a augmenté la proportion des lésions de petites tailles voire infra cliniques au diagnostic [10, 11]. Le curage ganglionnaire a été indiqué et réalisé pour 36 cas (94,73% des patientes opérées). L'examen histo-pathologique a été réalisée pour 34 pièces opératoires (89,47% des pièces opératoires). Le nombre moyen des ganglions du produit de curage était de 11,15. Ce qui est superposable aux résultats de JIHEN [14] en Tunisie, BIJEK [15] en France qui ont noté un nombre moyen de 11,8 et de 13 ganglions. Cela montre bien une maîtrise correcte de la technique du curage axillaire dans le centre hospitalier universitaire Yalgado Ouédraogo

Dans notre série, l'envahissement ganglionnaire a été noté dans 71,87% des cas. Le nombre moyen des ganglions envahis était de 5,22 (avec des extrêmes de 2 à 18). Cela est similaire à celui de DEM [12] au Sénégal qui rapportait un taux d'envahissement de 79% dans sa série. Notre taux est nettement supérieur à ceuxde Erdem en Inde (39,26%)^16^, JIHEN [14] en Tunisie (54,7%), BIJEK [15] en France (38,86%). Cela s'expliquerait sans doute par la taille des tumeurs au moment du diagnostic dans notre étude (T3 ou T4 dans 82,6% des cas). En effet, il existe une corrélation entre la taille de la tumeur et l'envahissement ganglionnaire [17]. Notre taux de lymphocèle était de 23,68%. En post opératoire, la fréquence d'apparition de lymphocèle est de l'ordre de 20 à 40% [18, 19]. La technique du ganglion sentinelle a permis de réduire d'un facteur 4 Le risque de lymphocèle [20].

En récapitulant les causes de survenu des complications, BIJEK [15] en France a démontré que le risque de survenu de lymphocèle augmentait avec le nombre de ganglions prélevé, le curage axillaire et l'envahissement ganglionnaire clinique ou histologique. L'utilisation de drain (drain aspiratif de REDON) [18], un dépistage précoce des lésions et l'acquisition de matériel adéquat permettant la technique du ganglion sentinelle, réduirait le risque de survenu de lymphocèle dans notre contexte de travail. La technique du ganglion sentinelle est indiquée pour des tumeurs uni focales inférieures à 2 cm sans envahissement axillaire.

## Conclusion

La chirurgie occupe le second rôle dans la prise en charge des cancers du sein lorsqu'une chirurgie est encore possible dans notre hôpital. Un dépistage précoce permettrait la réalisation d'un traitement conservateur d'emblée, une diminution du taux de complications en post opératoire. Cela contribuerait à une réduction du coût de la prise en charge.

## References

[1] Organisation Mondial de la Santé (OMS) Cancer.

[2] Organisation Mondial de la Santé (OMS) (2013). La santé des femmes. Centre des médias, aide-mémoire N°334.

[3] Bosetti C, Bertuccio P, Levi F, Chatenoud L (2012). The decline in breast cancer mortality in Europe: an update (to 2009). Breast..

[4] Siegle R, Desantis C, Virgo K (2012). Cancer Treatment and Survivorship Statistics, 2012. CA Cancer J Clin..

[5] Goncalves A, Tredan O, Villanueva C, Dumontet C (2012). Antibody-drug conjugates in oncology: from the concept to trastuzumabemtansine (T-DM1). Bull Cancer..

[6] Akram M, Siddiqui SA (2012). Breast cancer management: past, present and evolving. Indian J Cancer..

[7] Hiram S, Sara S (1982). The continuing importance of adequate surgery for operable breast cancer: significant salvage of node positive patients without adjuvant chemotherapy. CA Cancer J Clinvol.

[8] Ly M, Antoine M, Andre F, Callard P, Bernaudin JF, Diallo DA (2011). Le cancer du sein chez la femme de l'Afrique sub-saharienne. Bull Cancer..

[9] Honig A, Rieger L, Sutterlin M (2005). State of the art of neoadjuvant chemotherapy in breast cancer: rationale, results and recent developments. Ger Med Sci..

[10] Cutuli B, Lemanski C (2009). Breast-conserving surgery with or without radiotherapy vs mastectomy for ductal carcinoma in situ: French Survey experience. Br J Cancer..

[11] Greenberg CC, Lipsitz SR, Hughes ME, Edge SB (2011). Institutional variation in the surgical treatment of breast cancer: a study of the NCCN. Annals of surgery..

[12] Dem A, Dieng MM, Ndiaye Ba N, Gaye PM, Gaye-Fall MC, Diouf D (2011). Breast lobular carcinoma: 43 cases report. CarcinolClinAfrique..

[13] Damoaliga G (2009). Les difficultés diagnostiques et thérapeutiques des cancers du sein chez la femme au CHU-YO: à propos de 55 cas.

[14] Jihen J, Habib A, Nabil T, Sourour Y (2010). Le cancer du sein chez la femme âgée: épidemiologie et caractéristiques cliniques. J I M Sfax..

[15] Bijek JH, Aucouturier JS, Doridot V (2005). Lymphocèles axillaires après curage ou prélèvement du ganglion sentinelle en cas de cancer du sein. Bull Cancer..

[16] Erdem E, Alagol H (2009). Results of breast conserving surgery in clinical stage I-II breast carcinoma. Indian J Surg..

[17] Liu C, Pan H, Li Z, Shi L, Huang T (2009). Histopathological features of invasion of breast invasive ductal carcinoma and safety of breast-conserving surgery. J HuazhongUnivSciTechnolog Med Sci..

[18] Andeweg CS, Schriek MJ, Heisterkamp J, Roukema A (2011). Seroma formation in two cohorts after axillar y lymph node dissection in breast cancer surgery: does timing of drain removal matter?. The Breast Journal..

[19] Lumachi F, Brandes AA, Burelli P, Basso SM, Lacobonne M, Ermani M (2004). Seroma prevention following axillary dissection in patients with breast cancer by using ultrasound scissors: a prospective clinical study. Eur J SurgOncol..

[20] Mansel RE, Fallowfield L, Kissin M, Goyal A (2006). Randomized multicenter trial of sentinel node biopsy versus standard axillary treatment in operable breast cancer: the ALMANAC trial. J Natl Cancer Inst..

